# Comparison of apical debris extrusion using a conventional and two rotary techniques

**Published:** 2009-10-10

**Authors:** Alireza Adl, Safoora Sahebi, Fariborz Moazami, Mahnaz Niknam

**Affiliations:** 1 *Department of Endodontics, Dental School, Shiraz University of Medical Sciences, Shiraz, Iran.*; 2 *General Dentist, Shiraz, Iran*.

**Keywords:** Debris extrusion, FlexMaster, Mtwo, Root canal preparation, Step-back

## Abstract

**INTRODUCTION:** Preparation techniques and instruments produce and push debris out of canals. This can induce inflammation within the periapical area. Therefore, instrumentation that causes less extrusion of debris is more desirable. The purpose of this *in vitro* study was to evaluate the quantity of debris extruded from the apical foramen during root canal preparation by using one hand, and two rotary instrumentation techniques.

**MATERIALS AND METHODS:** Three different groups each with 12 mesiobuccal roots of human maxillary first molar were instrumented using either step-back technique with hand instruments, FlexMaster or Mtwo rotary system. Debris extruded from the apical foramen during canal preparation was collected. The mean dry weights of debris were compared using one-way ANOVA.

**RESULTS:** Step-back group had a significantly greater mean weight of debris compared to the other two groups (P<0.05). Mtwo group had the lowest mean weight of debris, though it was not significantly different from FlexMaster group.

**CONCLUSION:** According to this study, the engine driven techniques were associated with less apical debris extrusion. [Iranian Endodontic Journal 2009;4(4):135-8]

## INTRODUCTION

In asymptomatic chronic periradicular lesions, a delicate balance exists between infected canal microbiota and the host defenses. If quantities of bacteria are extruded apically during root canal preparation, this balance will be disrupted and an acute inflammatory response may ensue to re-establish the equilibrium (1). Therefore minimizing the apical extrusion of debris can minimize postoperative reactions.

Several studies have shown that all preparation techniques and instruments produce and push debris out of canals, even when instrumentation is confined in the root canal area (2-8).

During the last decade, root canal preparation with NiTi Rotary systems has become popular. More recently, instruments with non-cutting tips, radial lands, different cross-sections and varying tapers are available to improve working safety, reduce working time and create a greater flare within preparations (9).

A thorough comparison between new rotary systems and older techniques and the amount of debris they extrude as well as other parameters may be beneficial so that the best technique with the lowest incidence of post-operative flare up and extrusion may be selected.

Reddy and Hicks were the first who compared apical debris extrusion between hand instrumentation and engine-driven techniques (5). When comparing the mean weights of apically extruded debris, they noted that the step-back technique produced significantly more debris than the engine-driven and the balanced-force technique (5).

Hinrichs *et al.* showed that there were no significant differences between Lightspeed, Profile, NT McXIM and balanced force techniques and the amount of debris extruded (4). Ferraz *et al.* reported that engine-driven techniques (Profile 0.04, Quantec 2000 and Pow-R) extruded less debris than manual ones (6).

Zarrabi *et al.* compared the quantity of debris extruded from the apical foramen during canal preparation using three rotary systems (Profile, Race, and FlexMaster) and manual step-back technique. They concluded that the Race system induces less extruded debris than the manual technique and the FlexMaster system (10). However, another study showed no significant difference in the amount of debris extruded apically between manual technique and three rotary systems (K3, Race, FlexMaster) (11). Recently Mtwo a new rotary instrumentation system has been introduced which is based on a single length technique instead of crown down preparation. All instruments in this system reach the working length during canal preparation.

To date, there has been no literature published on the apical extrusion of debris during root canal preparation using Mtwo system. The purpose of this study was to compare *ex vivo* the amount of debris extruded apically, using Mtwo, FlexMaster and manual step-back technique.

## MATERIALS AND METHODS

Mesiobuccal roots of 36 extracted human maxillary first molar were used in this *in vitro* study. Buccal and proximal radiographic observations were performed to exclude teeth with calcification and open apices. The specimens were divided into 3 experimental groups of 12 roots each.

Debris and remnants of soft tissues around selected teeth were removed and teeth were cleaned and stored in 10% buffered formalin. Root curvatures were measured according to Schneider method (12). Teeth with curvature between 20 to 40 degrees were selected. Crowns were decoronated at the cementoenamel junction to achieve root lengths approximately 12±2 mm. Canal patency was controlled with K-file size #10 (Mani, Japan). Working length was determined 1 mm shorter than the length at which the file was visible through the apical foramen. Ten mL of distilled water with a 28 gauge needle (Supa, Tehran, Iran) was used for irrigation root canals. The three groups are outlined below.

Group 1 (manual technique group): K-files (Mani, Japan) were used with a primary quarter clockwise rotation followed by a pull-back motion until working length was reached. Apical preparation was performed up to file size #30 followed by step-back technique up to size #80. Group 2 (FlexMaster group): FlexMaster instruments (VDW, Munich, Germany) were used in a gear reduction handpiece (Endo IT professional, Aseptico Inc, USA) at the fixed speed of 280 rpm; in this group teeth were prepared with a crown down technique according to the manufacturer’s instructions using a gentle in-and-out motion. Instruments were withdrawn when resistance was felt and changed for the next smaller sized instrument. File sequences were used in the following sequence: size 0.06/20 was used one half of the working length; size 0.04/30 was used three quarters of working length; size 0.04/20 was used between three quarters of the working length and working length; and instruments of size 0.02/20, 0.02/25, and 0.02/30 were used to the working length.

Group 3 (Mtwo group): M2 instruments (VDW, Munich, Germany) were used in a gear reduction hand piece (Endo IT professional, Aseptico INC, USA) at the constant speed of 280 rpm Teeth in this group were prepared according to the protocol described by the manufacturer *i.e.* a single length technique. The instruments were used the following sequences, all to the working length: 0.04/10, 0.05/15, 0.06/20, 0.06/25, and 0.05/30. The methods used for debris collection was carried out as described by Myers and Montgomery (13)*.* Each root was forced through a rubber plug so that it could be easily held during instrumentation. The extruded debris and irrigants were collected in a pre-weighed receptor tube, attached to the lower edge of the rubber plug. The root apex was allowed to be hung within the receptor tube. A side-mouth bottle was used to hold the device during instrumentation. The bottle was vented with a 25-gauge needle (Supa, Tehran, Iran) alongside the rubber plug to unify the pressure inside and outside bottle. The bottle was obscured with a paper so that the operator was shielded from seeing the root apex during the instrumentation. Once instrumentation had been completed, each tooth was separated from the receptor tube and the debris adhering to the root surface was collected from root surface by washing the root with 2 mL of distilled water into the receptor tube. The receptor tubes were then stored in an incubator at 68˚C for 5 days in order for moisture to evaporate before weighing the dry debris. An electronic balance (Mettler Toledo, Bradford, USA) was used to weigh the debris. This was repeated until three consecutive identical weights were obtained for each sample. The mean dry weights of extruded debris were analyzed statistically using SPSS (version 13.0) for Windows (SPSS Inc., Chicago, IL, USA). The Kruskal-Wallis nonparametric test was applied to determine if significant differences existed between groups (P*<*0.05).

**Table 1:** Mean weight of extruded debris and SD using three different systems

Technique	Mean (mg)	SD
Manual	14.45	0.72
FlexMaster	0.39	0.41
Mtwo	0.34	0.28

## RESULTS

According to our results, all three techniques induced extrusion of debris from the apical foramen. One-Way ANOVA analysis indicated that there were significant differences between the three groups (Table 1). Group 1 (manual technique) had the highest mean weight of debris which was significantly different from two other groups (P<0.05). Group 3 (Mtwo) had the lowest mean weight of debris; though this was not significantly different from group 2 (FlexMaster).

## DISCUSSION

In this study the type and quantity of irrigants used were the same, instrumentations were performed by one operator, and the working length for all specimens was determined 1 mm shorter than the apical foramen, so that variables which may affect results could be minimized. Also, master apical file was the same in all groups.

Some authors have used NaOCl for irrigation (5,11,14,15), whereas others have used distilled water (2,3,6,10,16). Distilled water was used in this study to avoid any possible weight increase due to NaOCl crystal formation.

In our study engine-driven instruments extruded less debris than K-files for step-back technique. Kustarci *et al.* (11) compared Race, K3, FlexMaster, and step-back manual technique using K-file. The most apical extruded debris was observed in the manual technique, although no significant difference was observed among all groups. Zarrabi *et al.* (10) compared profile, Race, and FlexMaster instrument with the step-back technique and reported that the step-back technique extruded greater debris than rotary instruments. Ferraz *et al.* (6) reported that the profile instrument induced less extruded debris than manual technique. Azar and Ebrahimi reported that Protaper and Profile rotary instruments extruded less debris than the step-back (7), also Ruddy and Hicks showed that step-back instrumentation produced significantly more debris than rotary instrumentation and balanced force technique (5). Therefore, our results were similar to previous studies that showed that engine-driven instruments extended less debris than manual technique.

During step-back technique, the file acts as a plunger in the apical third to force the debris ahead of the file; this may be the reason for greater apical extrusion of debris.

In this study two rotary systems with different techniques were applied; that is the FlexMaster instruments that involved crown down technique, and the Mtwo instrument that were used single length method. The mean weight of debris produced in FlexMaster group and M2 group were almost equal.

According to findings of this study we can suggest that reduction of debris extrusion in rotary preparation techniques is not due to the crown down technique but rather related to rotational motion of files. A probable explanation for this finding is that rotary motion tends to pull dentinal debris into the flutes of the file and directs it toward the coronal aspect of the canal (3).

The results of this study are consistent with Reddly and Hicks (5) that suggested rotational motion was associated with less debris extruded apically when compared with a push-pull filing technique.

The results of present *in vitro* study may be repeated through *in vivo* experiments. Periodontal and granulation tissues of chronic apical lesion may act as a natural barrier and prevent apical debris extrusion *in-vivo*. Also, studies using hand balance forced technique should also be compared.

## CONCLUSION

The engine-driven techniques extruded less debris compared to step-back technique, presumably due to the rotary motion, which tents to direct debris towards the coronal orifice, avoiding its compaction in the root canal.
